# Levetiracetam enhances the temozolomide effect on glioblastoma stem cell proliferation and apoptosis

**DOI:** 10.1186/s12935-018-0626-8

**Published:** 2018-09-10

**Authors:** Bianca Maria Scicchitano, Silvia Sorrentino, Gabriella Proietti, Gina Lama, Gabriella Dobrowolny, Angela Catizone, Elena Binda, Luigi Maria Larocca, Gigliola Sica

**Affiliations:** 1 Istituto di Istologia ed Embriologia, Università Cattolica del Sacro Cuore, Fondazione Policlinico Universitario A. Gemelli IRCCS, Roma, Italia; 2 DAHFMO-Unit of Histology and Medical Embryology, Sapienza University of Rome, Via Scarpa 16, 00161 Rome, Italy; 3 ISBReMIT-Cancer Stem Cells Unit, IRCSS Casa Sollievo della Sofferenza, Viale Padre Pio 7, 71013 San Giovanni Rotondo, FG Italy; 4 Istituto di Anatomia Patologica, Università Cattolica del Sacro Cuore, Fondazione Policlinico Universitario A. Gemelli IRCCS, Roma, Italia

**Keywords:** Glioblastoma, Cancer stem cells, MGMT, Temozolomide, Levetiracetam

## Abstract

**Background:**

Glioblastoma multiforme (GBM) is a highly aggressive brain tumor in which cancer cells with stem cell-like features, called cancer stem cells (CSCs), were identified. Two CSC populations have been previously identified in GBM, one derived from the GBM area called enhanced lesion (GCSCs) and the other one from the brain area adjacent to the tumor margin (PCSCs) that greatly differ in their growth properties and tumor-initiating ability. To date the most effective chemotherapy to treat GBM is represented by alkylating agents such as temozolomide (TMZ), whose activity can be regulated by histone deacetylases (HDACs) inhibitors through the modulation of O6-methylguanine-DNA methyltransferase (MGMT) expression. Levetiracetam (LEV), a relatively new antiepileptic drug, modulates HDAC levels ultimately silencing MGMT, thus increasing TMZ effectiveness. However, an improvement in the therapeutic efficacy of TMZ is needed.

**Methods:**

Cell proliferation was investigated by BrdU cell proliferation assay and by Western Blot analysis of PCNA expression. Apoptosis was evaluated by Western Blot and Immunofluorescence analysis of the cleaved Caspase-3 expression. MGMT and HDAC4 expression was analyzed by Western Blotting and Immunofluorescence. Statistical analysis was performed using the Student’s *t* test and Mann–Whitney test.

**Results:**

Here we evaluated the effect of TMZ on the proliferation rate of the IDH-wildtype GCSCs and PCSCs derived from six patients, in comparison with the effects of other drugs such as etoposide, irinotecan and carboplatin. Our results demonstrated that TMZ was less effective compared to the other agents; hence, we verified the possibility to increase the effect of TMZ by combining it with LEV. Here we show that LEV enhances the effect of TMZ on GCSCs proliferation (being less effective on PCSCs) by decreasing MGMT expression, promoting HDAC4 nuclear translocation and activating apoptotic pathway.

**Conclusions:**

Although further studies are needed to determine the exact mechanism by which LEV makes GBM stem cells more  sensitive to TMZ, these results suggest that the clinical therapeutic efficacy of TMZ in GBM might be enhanced by the combined treatment with LEV.

**Electronic supplementary material:**

The online version of this article (10.1186/s12935-018-0626-8) contains supplementary material, which is available to authorized users.

## Background

Glioblastoma multiforme (GBM) is one of the most aggressive primary brain tumor. It has been demonstrated that mutations of the IDH1 gene correlate with outcome in patients with malignant glioma and are considered as independent factors for predicting longer overall survival and progression free survival in patients with GBM [1]. In GBM, cancer cells with stem cell-like features, called cancer stem cells (CSCs), were identified [2, 3]. GBM stem-like cells (GSCs) have a high capacity to resist or to adapt to standard therapies which include surgery followed by radiotherapy and chemotherapy [4–6], resulting in a poor prognosis with a median survival time of about 14 months [7, 8]. Thus, the development of efficient strategies targeting these cells  is urgently needed.

It has been demonstrated that CSC distinct pools reside within different regions of the same GBM [9–11]. More recently, the presence of two GSC populations, one derived from the GBM area called enhanced lesion (GCSCs) and the other one from the brain area adjacent to the tumor margin (PCSCs) that greatly differ in their growth properties and tumor-initiating ability,  was identified. Indeed, GCSCs and PCSCs possess key neural stem cell features, such as multipotency, clonogenic ability and extensive self-renewal, together with aberrant growth properties [12]. Moreover, the area adjacent to the tumor shows edema, vascular alterations [13, 14], reactive astrocytes and microglia [15, 16] in addition to an abnormal gene expression  [17, 18]. It has been demonstrated that tumor recurrence occurs in tissue neighboring GBM in approximately 90% of patients, suggesting a growing relevance for this area in translational research [16, 19].

GSCs can be isolated from surgical specimens through mechanical dissociation of the tumor tissue and culture in a serum-free medium. In this conditions GCSC- and PCSC-derived cell clones are able to grow in vitro in aggregates called neurospheres and maintain an undifferentiated state as demonstrated by the expression of stem cell markers. Moreover, when injected in immunosuppressed mice, these cells are able to generate a tumor identical to the original one in terms of antigen expression and histological features, although GCSCs exhibit a higher tumor-initiating ability and clonogenicity when compared with PCSCs [12]. For all the above, GCSCs and PCSCs represent a good model to study glioblastoma response to treatments and, in particular, to highlight the role of neighboring microenvironment in tumor progression. To date the most effective chemotherapies to treat GBM are alkylating agents such as Temozolomide (TMZ), with a good penetration into the blood–brain barrier [20]. Alkylating agents damage DNA by formation of different small and bulky adducts with nucleic acid bases. In particular, TMZ acts by delivering a methyl group to purine bases of DNA leading to cell cycle arrest and, eventually, to apoptosis [21]. O6-Methylguanine-DNA-methyltransferase (MGMT) repairs the most cytotoxic lesions generated by TMZ, by removing the methyl adducts from DNA. MGMT promoter methylation, leading to a transcriptional silencing, correlates with improved survival in GBM patients exposed to alkylating agent treatment. Accordingly, expression of MGMT is one of the most robust predictors of the TMZ response in malignant glioma cells [22–24]. Epigenetic mechanisms are increasingly recognized as a major factor contributing to pathogenesis of cancer including glioblastoma. Enzymatic modification of histone proteins regulating gene expression are recently being exploited for therapeutic drug targeting. In particular, histone acetylation and deacetylation have been demonstrated to regulate several physiological and pathological cellular processes. Histone acetylation is mediated by histone acetyltransferases (HATs) and generally allows for active gene transcription. Conversely, histone deacetylation is catalyzed by histone deacetylases (HDACs) and favors gene repression. Eighteen distinct HDACs have been identified so far and they are classified into four classes based on their sequence and catalytic activity. HDAC4 is a member of class II and it has been demonstrated to be involved in progression of GBM [25]. In addition, it has been recently  reported that a great number of non-histone proteins can undergo reversible acetylation by HATs and HDACs. Modifications in this dynamic equilibrium can disturb cell homeostasis and result in a pathological state [26]. Nevertheless, HDAC inhibitors cause acetylation of both histone and non-histone proteins and exert multiple anti-tumoral effects by inducing differentiation, apoptosis, cell cycle arrest, susceptibility to chemotherapy and inhibition of migration and angiogenesis [27].

Emerging evidences demonstrate that some antiepileptic drugs (AEDs) have a transcriptional regulatory activity via HDAC modulation [28]. In particular, it has been shown that Levetiracetam (LEV), a relatively new AED, increases the transcription of HDACs and recruits corepressor complex on MGMT promoter, thus silencing its activity [22].

In this work, we used the GCSC and the PCSC neurospheres derived from primary GBM of six patients, according to the WHO 2016 classification, and we evaluated the effect of several antineoplastic drugs such as TMZ, Etoposide (ETO), Irinotecan (IRI) and Carboplatin (CARB) on their proliferation. Since our results demonstrated that the chemotherapeutic agent with less efficacy was TMZ, we subsequently investigated the possibility to increase its cytotoxic activity by the concomitant treatment with LEV.

Here we show that LEV enhances the effect of TMZ on GCSCs proliferation (being less effective on PCSCs) by decreasing MGMT expression, promoting HDAC4 nuclear translocation and activating apoptotic pathway. Although further studies are needed to determine the exact mechanism by which LEV sensitizes GBM stem cells to TMZ, these results suggest that the clinical therapeutic efficacy of TMZ in GBM might be enhanced by the combination treatment with LEV.

## Materials and methods

### Neurosphere culture

A total of six pairs of neurospheres derived from both GBM and peritumoral tissue (at a distance < 1 cm from macroscopic tumor border), called Glioblastoma Cancer Stem Cells (GCSCs) and Peritumoral Cancer Stem Cells (PCSCs), respectively, were kindly provided by Vescovi and Binda. According to the classification of human gliomas by WHO [29], we analyzed the GCSC and PCSC neurospheres for the mutational status of IDH1 [30, 31]. Mutational IDH1/2 status and MGMT promoter methylation status were also investigated in all the available tissue samples from which the neurospheres were derived [32].

The neurospheres were cultured in NeuroCult™ NS-A Proliferation Kit (StemCell Technologies Inc, Vancouver, BC, Canada) supplemented with 20 ng/mL human recombinant EGF, 10 ng/ml human recombinant bFGF and 2 µg/mL heparin (all from StemCell Technologies Inc.), as described previously [33]. All cell cultures were maintained at 37 °C in a 5% CO2 humidified atmosphere.

### IDH analysis

IDH1 was amplified from 20 ng genomic DNA with forward primer 5′- ACCAAATGGCACCATACGA-3′ and reverse primer 5′- TTCATACCTTGCTTAATGGGTGT-3′ using conditions described by Balss J. et al. [34]. The resulting PCR products were sequenced using the ABI Prism BigDye Terminator v3.1 Cycle Sequencing Kit (Applied Biosystems, 4337455). Thirty cycles were performed employing 10 mM of the sense primers with denaturing at 95 °C for 10 s, annealing at 56 °C for 5 s and extension at 60 °C for 240 s. A second round of sequencing analysis was performed using the antisense primer. Sequences were determined using the automated AB3130xl (Applied Biosystems, CA, USA). Results were analyzed with Chromas Lite software (Technelysium) and Mutation Surveyor software (SoftGenetics).

### O6-Methylguanine-DNA methyltransferase (MGMT) promoter methylation analysis

O6-methylguanine-DNA methyltransferase (MGMT) promoter methylation patterns were studied by methylation-specific PCR using primers specific for methylated and unmethylated DNA [35] on genomic DNA extracted from paraffin-embedded tissue using QIAamp DNA Mini kit (Qiagen). The annealing temperature was 60 °C. DNA from normal lymphocytes treated with SssI methyltransferase (New England Biolabs) was used as a positive control for methylated and unmethylated alleles of MGMT. PCR products were separated onto 3% agarose gel, stained with ethidium bromide, and visualized under UV illumination.

### Chemotherapeutic drugs and adjuvant molecules

Temozolomide, ETO and IRI (Sigma-Aldrich, Saint Louis, MO, USA) were dissolved in dimethyl sulfoxide (DMSO) (Sigma-Aldrich) at the concentration of 100 mM, 30 mg/mL and 10 mg/mL, respectively, while CARB and LEV (Sigma-Aldrich) were dissolved in deionized water at the concentration of 10 mg/mL and 5 mg/mL, respectively. All the drugs were stored as stock solutions at -80 °C and diluted in stem cells culture medium just before use. The final drug concentrations used in the experiments were: TMZ: 250 µM; ETO: 10 µM; IRI: 10 µg/mL and CARB: 10 µg/mL; LEV: 40 µg/mL.

When the cells were treated with both TMZ and LEV, LEV was added 2 h before TMZ addition and the cultures were stopped after 48 h. The chemotherapeutic drug concentrations utilized in our experiments are in the higher range of serum peak levels transiently reached in vivo during high-dose chemotherapy.

### BrdU cell proliferation assay

**Fig. 1:**
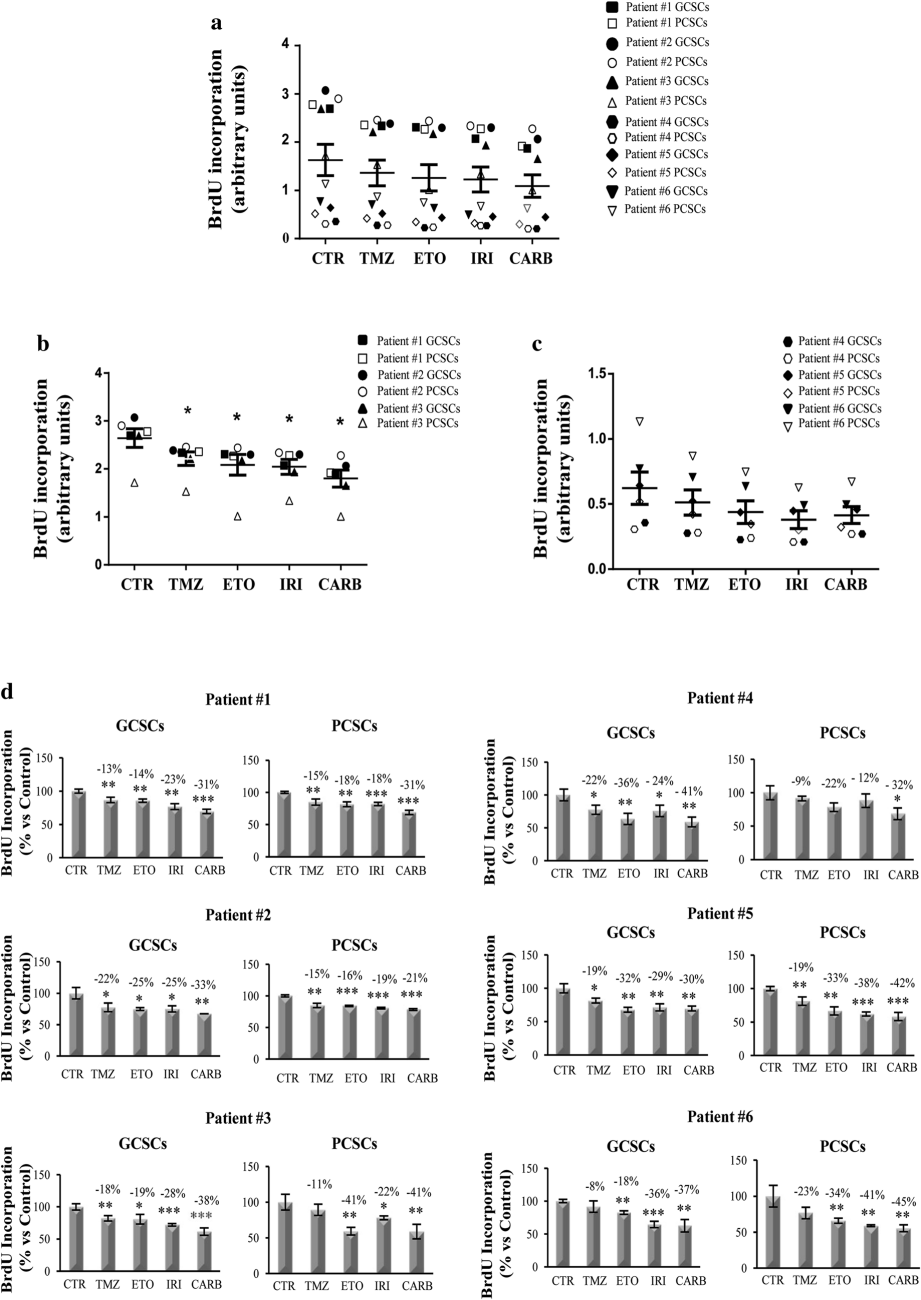
Effects of chemotherapeutic treatments on patient-derived GSC proliferation. GCSC and PCSC neurospheres derived from GBM of six different patients were treated with TMZ (250 µM), ETO (10 µM), IRI (10 µg/mL) and CARB (10 µg/mL) for 48 h. GCSCs and PCSCs proliferation rate was evaluated by BrdU incorporation. **a** The graph represents the distribution values of BrdU incorporation of the all neurospheres analyzed. **b** The graph represents BrdU incorporation of the GCSCs and PCSCs derived from patients #1, #2 and #3, that exhibit a higher capacity of BrdU incorporation. **c** The graph represents BrdU incorporation of the GCSCs and PCSCs derived from patients #4, #5 and #6, that exhibit a lower capacity of BrdU incorporation. K-means algorithm was used to cluster into 2 groups the BrDU incorporation values. **d** Percentage of decreased BrdU incorporation versus CTR. The results shown are representative of three independent experiments. **a**–**c** *p < 0.05 vs CTR by Mann–Whitney test. **d** * p< 0.05, ** p< 0.01, ***p < 0.001 vs CTR by Student’s *t*-test

GCSCs and PCSCs were seeded at the density of 50.000 cells/well in 96-well plates and incubated overnight. The cells were then treated with the appropriate chemotherapeutic agents, as indicated in the Fig. 1 for 48 h. Finally 10 µM BrdU was added to the plates and the cells were incubated overnight. BrdU proliferation assay was performed according to the manufacturer’s instructions (Cell Signaling, #6813 Danvers, MA, USA).

### Western blot analysis

For immunoblotting analysis, cells were washed in 1 × PBS, harvested and lysed in 1x Cell Lysis Buffer (Cell Signaling #9803) containing 1 mM PMSF (Cell Signaling #8553) and a complete protease inhibitor cocktail (Cell Signaling #5872) for 30 min at 4 °C. Then the cells were sonicated briefly and the extracts were centrifuged 10 min at 14,000 × *g* in a cold microfuge. Protein concentration was determined by Bradford Protein Assay (Bio-Rad Laboratories Inc, Hercules, CA, USA) according to the manufacturer’s instructions. Equal amounts of proteins were then separated by SDS/PAGE (Mini-PROTEAN^®^ TGX™ Precast Protein Gels, or Mini-PROTEAN TGX stain-free precast PAGE gels, Bio-Rad Laboratories Inc.) and transferred to a nitrocellulose membrane (GE Healthcare, Piscataway, NJ, USA). Membranes were blocked with Tris-buffered saline (TBS) 1X (Bio-Rad Laboratories Inc.) supplemented with 0.1% Tween-20 and containing 5% nonfat milk for 1 h at room temperature (RT). The primary antibodies used in this work  were: anti-MGMT (1:500, mouse monoclonal antibody, clone MT3.1, MAB16200, Merk Millipore, Darmstadt, Germany); anti-HDAC4 (1:100, rabbit monoclonal antibody, sc-46672 Santa Cruz Biotechnology, Dallas, Tx, USA); anti-PCNA (1:1000, mouse monoclonal antibody, M0879, Dako, Santa Clara, CA, USA); anti-cleaved Caspase-3 (1:1000, polyclonal antibody, #9665, Cell Signaling); anti-β-actin, (1:10000 mouse monoclonal antibody, Sigma-Aldrich). Blots were then incubated with horseradish peroxidase-conjugated secondary antibody (1:10,000, Vector Laboratories, Burlingame, CA, USA) for 1 h RT. Signals were captured by ChemiDoc™ Imaging System (Bio-Rad Laboratories, Hercules, CA, USA) using an enhanced chemiluminescence system (SuperSignal Chemoluminescent substrate, Thermo Fisher Scientific Inc. Waltham, MA, USA) and densitometric analyses were performed with Image Lab™ Touch Software (Bio-Rad Laboratories). Nuclear and cytosolic fractions were normalized using stain free technology (Bio-Rad Laboratories Inc.). All experiments were carried out in triplicate and representative results are shown.

### Immunofluorescence and confocal microscopy analysis

Immunofluorescence analysis was performed on GCSCs and PCSCs collected onto a glass slide using a Cytospin centrifuge (Shandon Centrifuge, Model Cytospin 3, Marshall Scientific, Hampton, NH, USA), fixed with 4% paraformaldehyde for 20 min, incubated with 0,01% Triton X-100 for 7 min and blocked with Super Block solution (UCS Diagnostic S.r.l., Morlupo, Italy) for 5 min. The slides were incubated overnight at 4 °C with the primary antibodies against: MGMT (1:100, Merk Millipore), HDAC4 (1:100; Santa Cruz Biotechnology, INC.) and cleaved Caspase-3 (1:400, Cell Signaling). The next day, the slides were incubated with the following secondary antibodies for 1 h at RT: Alexa Fluor 584 (1:1000, Invitrogen Molecular Probes, Eugene, OR, USA) and Alexa Fluor 488 (1:1000, Invitrogen Molecular Probes). The cells were cover-slipped with ProLong Gold antifade reagent with DAPI (Life Technologies) and examined with a confocal laser scanning microscope (TCS-SP2, Leica Microsystems, GmbH, Wetzlar, Germany) equipped with an Ar/ArKr laser and a HeNe lasers. The images were recovered utilizing the Leica Confocal software. Laser line was at 488 nm and 543 for alexafluor 488 and alexafluor 568 excitation, respectively. For each analyzed field, optical spatial series each composed of about 10 optical sections with a step size of 1 μm were obtained. The images were scanned under a 40× oil. In each experiment, negative controls without the primary antibody were included to check for nonspecific staining.

### Statistical analysis

Each experiment was repeated three times. Data are presented as the mean ± SD. Statistical analysis was generally performed using Student’s *t*-test, assuming equal variance, and p-values were calculated based on the 2-tailed test. A p-value of < 0.05 was considered statistically significant. The Mann-Whitney test was used for the analysis of distribution values of BrdU incorporation in neurospheres.

## Results

### TMZ was the less effective chemotherapeutic agent in decreasing the GCSC and PCSC proliferation

The analysis of the IDH1 status in all the GCSC and PCSC pairs derived from six patients revealed that all of them are IDH1-wildtype. Moreover, the analysis of mutational IDH1/2 status and MGMT promoter methylation status in all the available tissue samples revealed that all of them were IDH1/2 wildtype and showed an un-methylated MGMT status.

We then investigated the effects of different chemotherapeutic agents on the proliferation of the neurospheres isolated from the GBM derived from six different patients. For this purpose, GCSCs and PCSCs were treated with TMZ, ETO, IRI and CARB for 48 h and then the rate of proliferating cells was evaluated by BrdU incorporation. At first, we noted that when the data regarding GCSCs and PCSCs derived from all the patients were analyzed together they were distributed in two clusters: one with the higher and one with lower values of BrdU incorporation (Fig. 1a). Interestingly, all the chemotherapeutic agents exerted significant effects only in the cells with higher values of BrdU incorporation (GCSCs and PCSCs derived from patients #1, #2 and #3) (Fig. 1b), whereas none of the drugs affected significantly the cells with a lower proliferation rate (GCSCs and PCSCs derived from patients #4, #5 and #6) (Fig. 1c). We then evaluated, more specifically, the percentage of proliferative reduction induced by the chemotherapeutic agents when the GCSCs and the PCSCs derived from the GBM of each patient were considered separately (Fig. 1d). As expected, both GCSCs and PCSCs obtained from the GBM of all the patients exhibited a higher resistance in terms of proliferation to the chemotherapeutic drug treatments when compared to Jurkat cells that, as hematopoietic cells, represent a good general model to test chemotherapeutic agent activity [36] (Additional file 1 and Additional file 2: Figure S1). Although no significant differences were observed in the response between GCSCs and PCSCs, our results showed that, similarly to what happens in Jurkat cells, TMZ had the lower effect, as demonstrated by the percentage of decreased proliferative range (8–23% versus CTR), while CARB had the stronger effect since the percentage of decreased proliferative range was 21–45% versus CTR (Fig. 1d).

### Levetiracetam sensitized GSCs to TMZ treatment

**Fig. 2:**
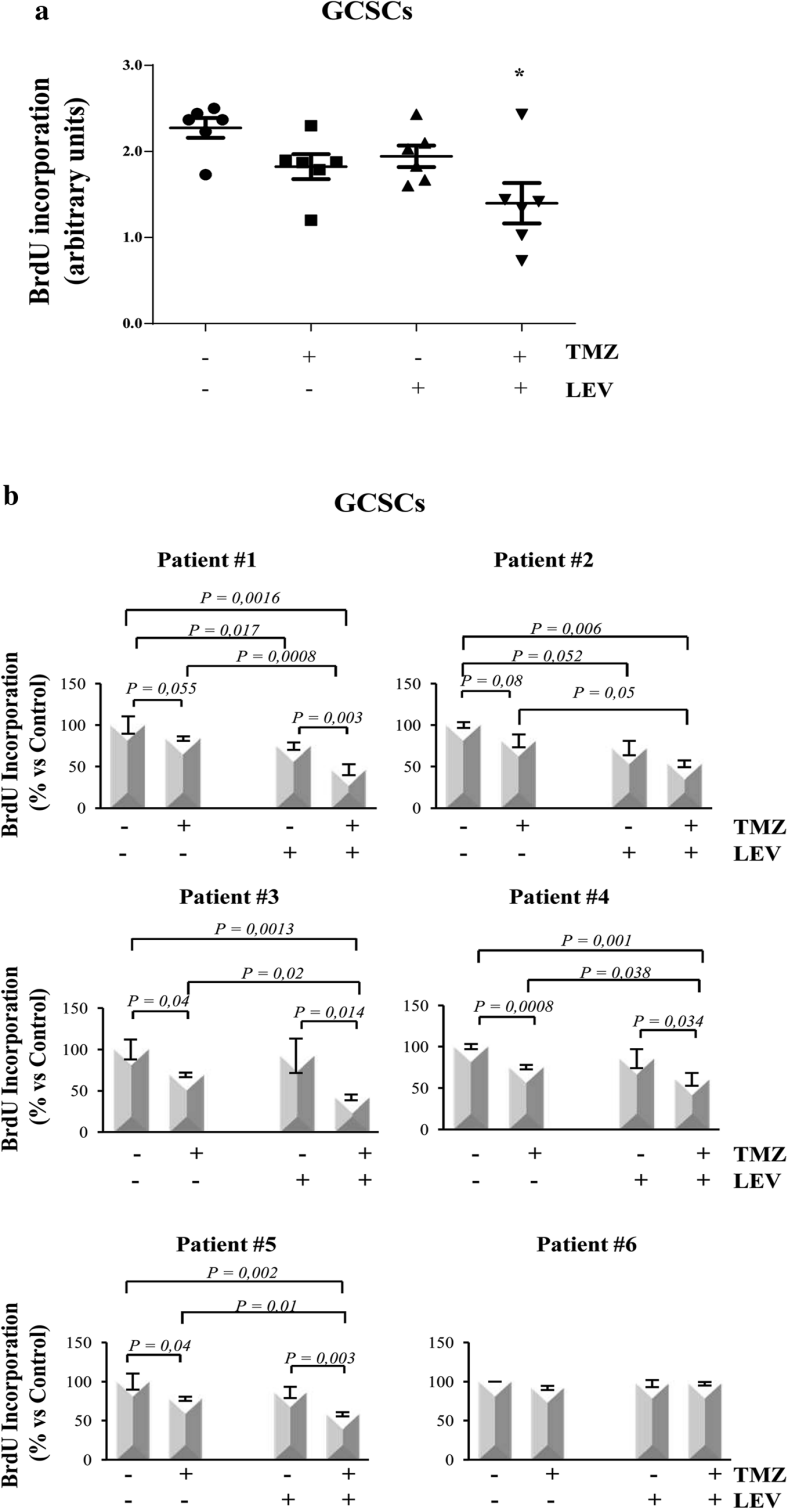
Effect of TMZ and LEV combined treatment on GCSC proliferation. GCSC neurospheres derived from GBM of six different patients were treated with TMZ (250 µM), LEV (40 μg/mL) or with a combination of TMZ and LEV for 48 h. Cell proliferation was evaluated by BrdU incorporation. The results were representative of three independent experiments. Statistical analysis was performed using Mann–Whitney test *p < 0.05 (**a**) and Student’s *t*-test (**b**). P value < 0.05 was considered statistically significant

**Fig. 3:**
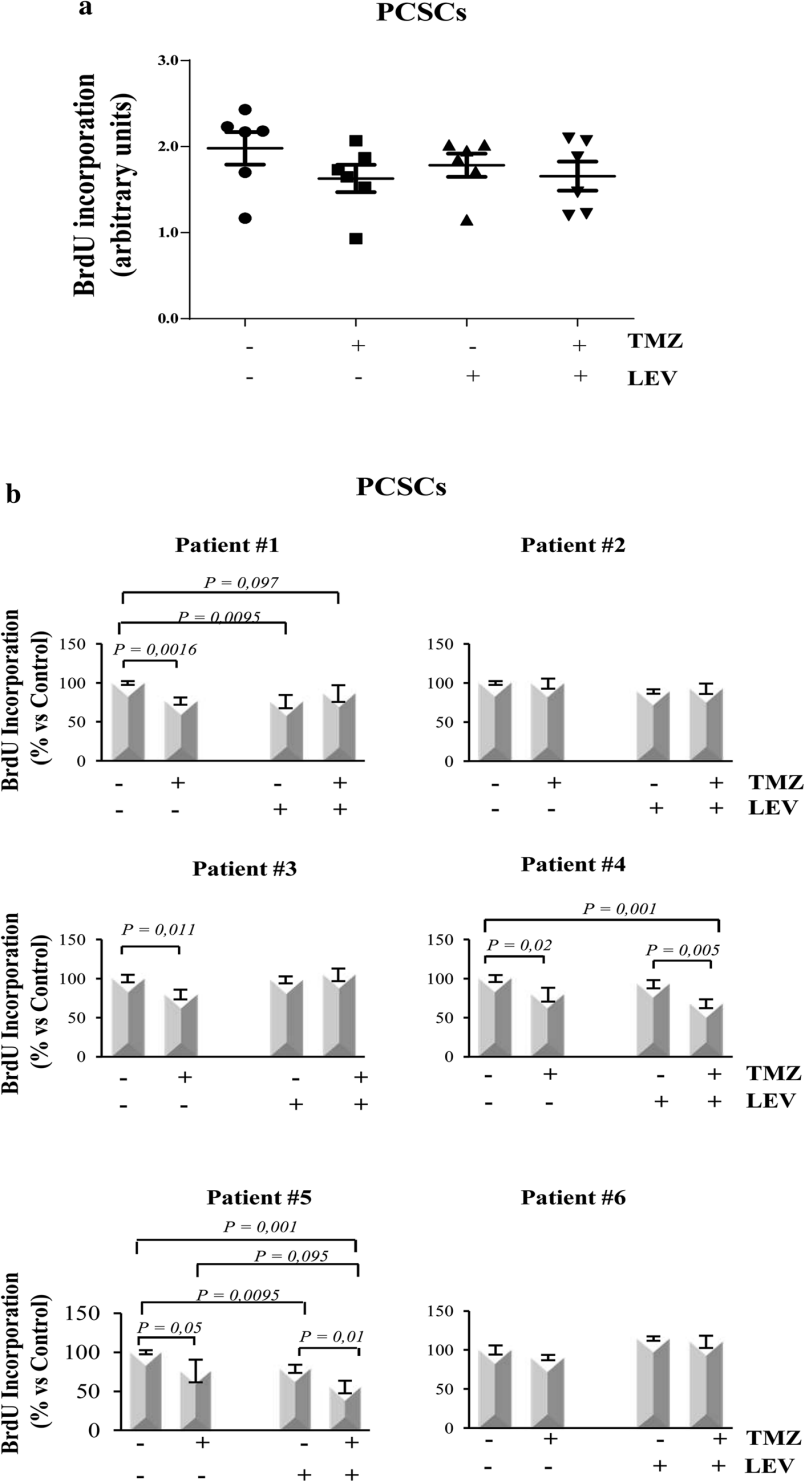
Effect of TMZ and LEV combined treatment on PCSC proliferation. PCSC neurospheres derived from GBM of six different patients were treated with TMZ (250 µM), LEV (40 μg/mL) or with a combination of TMZ and LEV for 48 h. Cell proliferation was evaluated by BrdU incorporation. The results were representative of three independent experiments. Statistical analysis was performed using Mann–Whitney test *p < 0.05 (**a**) and Student’s *t*-test (**b**). P value < 0.05 was considered statistically significant

Since our previous results demonstrated that, among the tested antineoplastic drugs, TMZ was the less efficient to decrease the proliferation of both GCSCs and PCSCs, we then investigated whether its anti-proliferative effect might be strengthened when combined with the LEV. LEV concentration used in this study (40 μg/mL) is included in the clinical serum therapeutic concentration range achieved in patients at oral doses of 500-1000 mg twice daily [28]. GCSCs and PCSCs isolated from the GBM derived from six patients were treated with TMZ, LEV or with a combination of TMZ and LEV, for 48 h. Figure 2a shows that, when analyzed together, the GCSCs derived from all the patients displayed a significant reduction of BrdU incorporation only with the combined treatment with TMZ and LEV. This result is confirmed by the analysis of BrdU incorporation evaluated in the GCSCs derived from each patient (Fig. 2b), demonstrating that the slight antitumor effect exerted by the treatment with TMZ or LEV alone was strongly enhanced when TMZ and LEV were added in combination. Only the GCSCs derived from patient #6 did not display a significant reduction in cell proliferation (Fig. 2b). Interestingly, the PCSCs subjected to the same treatments (Fig. 3a, b) seemed to be more resistant than the GCSCs since only the PCSCs derived from patients #4 and #5 (Fig. 3b) showed a significant decrease of proliferation when exposed to the combination of TMZ and LEV compared to either untreated cells or treated with these drugs alone. These results demonstrate that LEV sensitized GBM stem cells to TMZ and that this effect is stronger in GCSCs than in PCSCs.

### MGMT expression was downregulated in GCSCs treated with LEV + TMZ

**Fig. 4:**
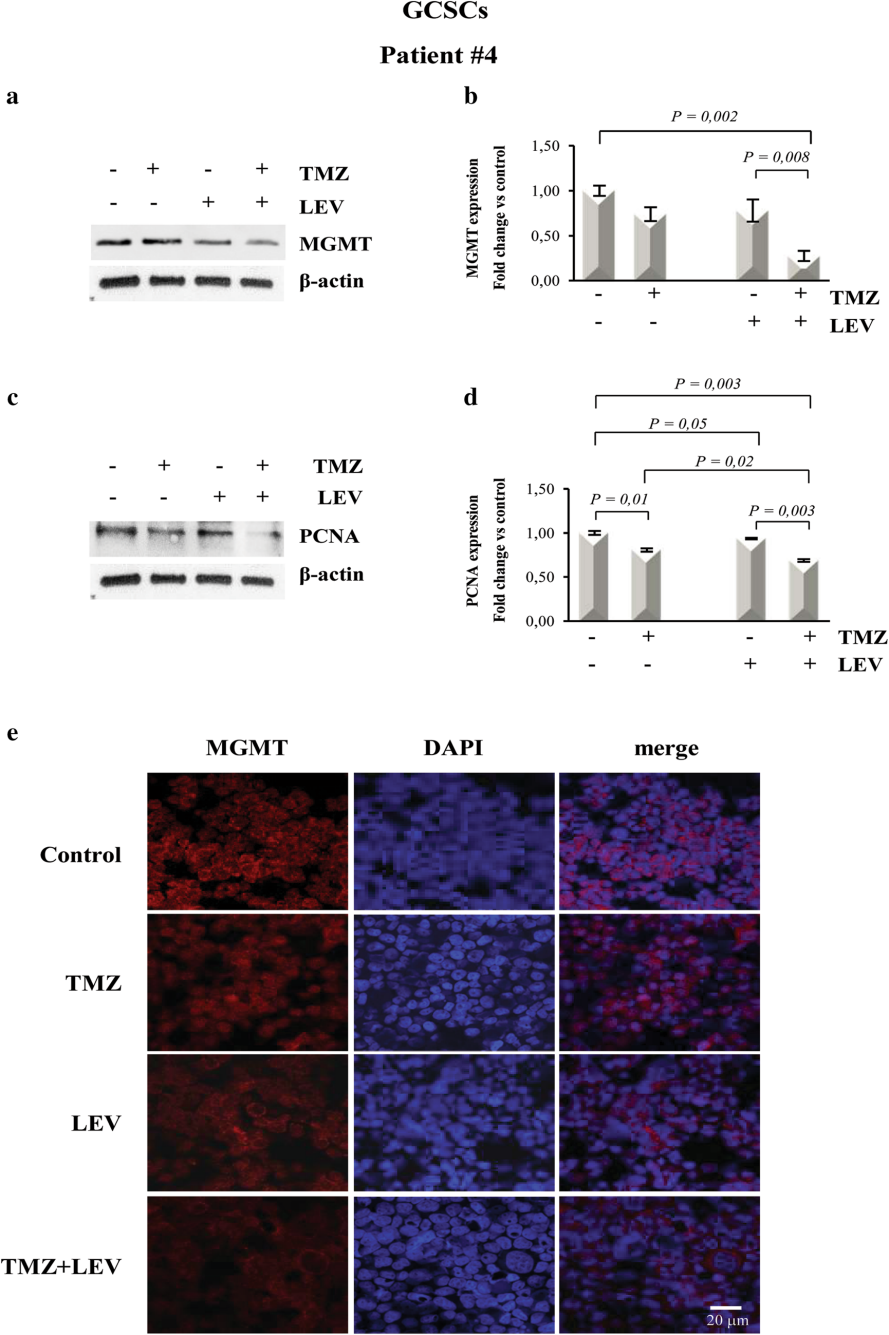
LEV effect on MGMT expression in GCSCs. GCSCs derived from patient #4 were treated with TMZ (250 µM), LEV (40 μg/mL) and with a combination of TMZ and LEV for 48 h. **a**, **c** Western blot analysis of MGMT and PCNA expression levels; representative immunoblots are shown. **b**, **d** Densitometric analysis of MGMT and PCNA expression levels (normalized to β-actin levels) of three independent experiments. Statistical analysis was performed using Student’s *t*-test. P value < 0.05 was considered statistically significant. **e** Confocal microscopy micrographs of GCSCs from patient #4 showing MGMT expression. Nuclei were stained with 4′,6-diamidino-2-phenylindole (DAPI). Original magnification: ×400

**Fig. 5:**
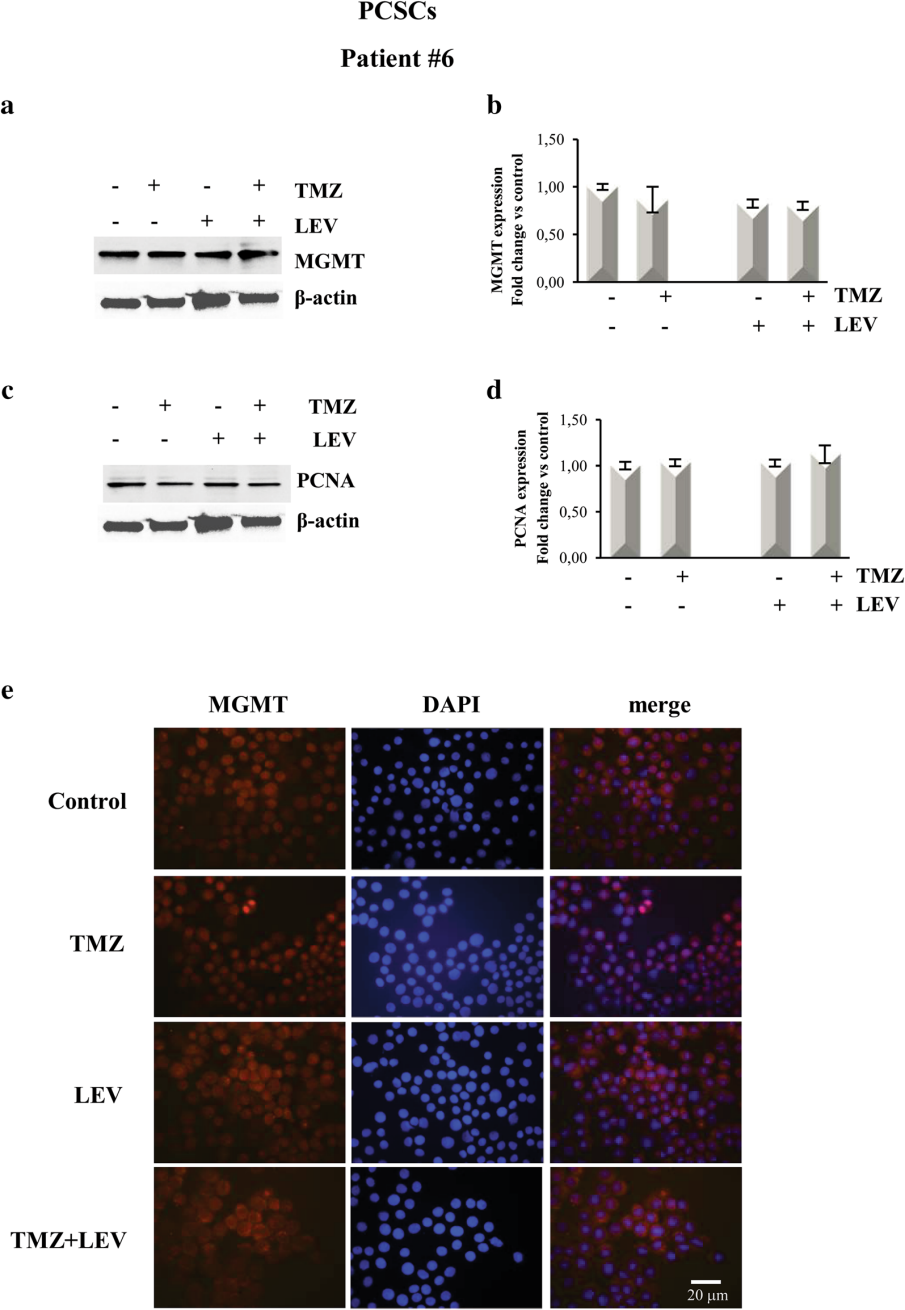
LEV effect on MGMT expression in PCSCs. PCSCs derived from patient #6 were treated with TMZ (250 µM), LEV (40 μg/mL) and with a combination of TMZ and LEV for 48 h. **a**, **c** Western blot analysis of MGMT and PCNA expression levels; representative immunoblots are shown. **b**, **d** Densitometric analysis of MGMT and PCNA expression levels (normalized to β-actin levels) of three independent experiments. Statistical analysis was performed using Student’s *t*-test. P value < 0.05 was considered statistically significant. **e** Confocal microscopy micrographs of PCSCs from patient #4 showing MGMT expression. Nuclei were stained with 4′,6-diamidino-2-phenylindole (DAPI). Original magnification: X 400

To test the hypothesis that LEV-induced sensitization to TMZ might result from the inhibition of MGMT-mediated DNA repair, we investigated MGMT protein levels. This analysis was performed in the GCSCs derived from patient #4, which displayed a high reduction of the proliferative rate after exposure to the combined treatment with LEV + TMZ and in PCSCs derived from patient #6, which showed resistance to all the treatments. The data obtained by BrdU cell proliferation assay were confirmed by Western blot analysis shown in Figs. 4c, d and 5c, d that demonstrated a decreased expression of the proliferation marker PCNA in the GCSCs of patient #4 when LEV and TMZ were added together, while its expression was not affected in the PCSCs derived from patient #6. Western blot analysis revealed a high level of MGMT expression in untreated GCSCs derived from patient #4; this expression was slightly decreased after treatment with TMZ and LEV singularly but it was dramatically decreased after the combined treatment with TMZ and LEV (Fig. 4a, b). In contrast, none of the treatments seemed to modify the MGMT expression level in the PCSCs derived from patient #6 when compared with untreated cells (Fig. 5a, b). Immunofluorescence analysis confirmed that the high expression of MGMT in control GCSCs derived from patient #4 (Fig. 4e) was partially decreased in the presence of either LEV or TMZ, while it was strongly reduced by the combined treatment with TMZ and LEV (Fig. 4e). In contrast, none of the treatments modified MGMT expression in PCSCs derived from the GBM of patient #6 (Fig. 5e). The PCNA and MGMT expression levels were also investigated in the PCSCs derived from patient #2 that similarly to the PCSCs of patient #6 did not show a reduction of the proliferative rate after exposure to all the treatments. Western blot analysis revealed that none of the treatments significantly affected the MGMT expression level, and the slight effect of TMZ and LEV on PCNA expression was not significantly modified by the combined treatment with TMZ + LEV (data not shown).

### LEV induced HDAC4 nuclear translocation in TMZ-treated GCSCs but not in PCSCs

It is has been demonstrated that some antiepileptic drugs, such as LEV, are able to regulate the HDACs activity. In particular, HDACs inhibitors can influence TMZ efficacy by modulating the expression of MGMT. Hence, we verified whether in our system, the LEV-induced sensitization to TMZ might be the result of a HDACs-dependent mechanism.

**Fig. 6:**
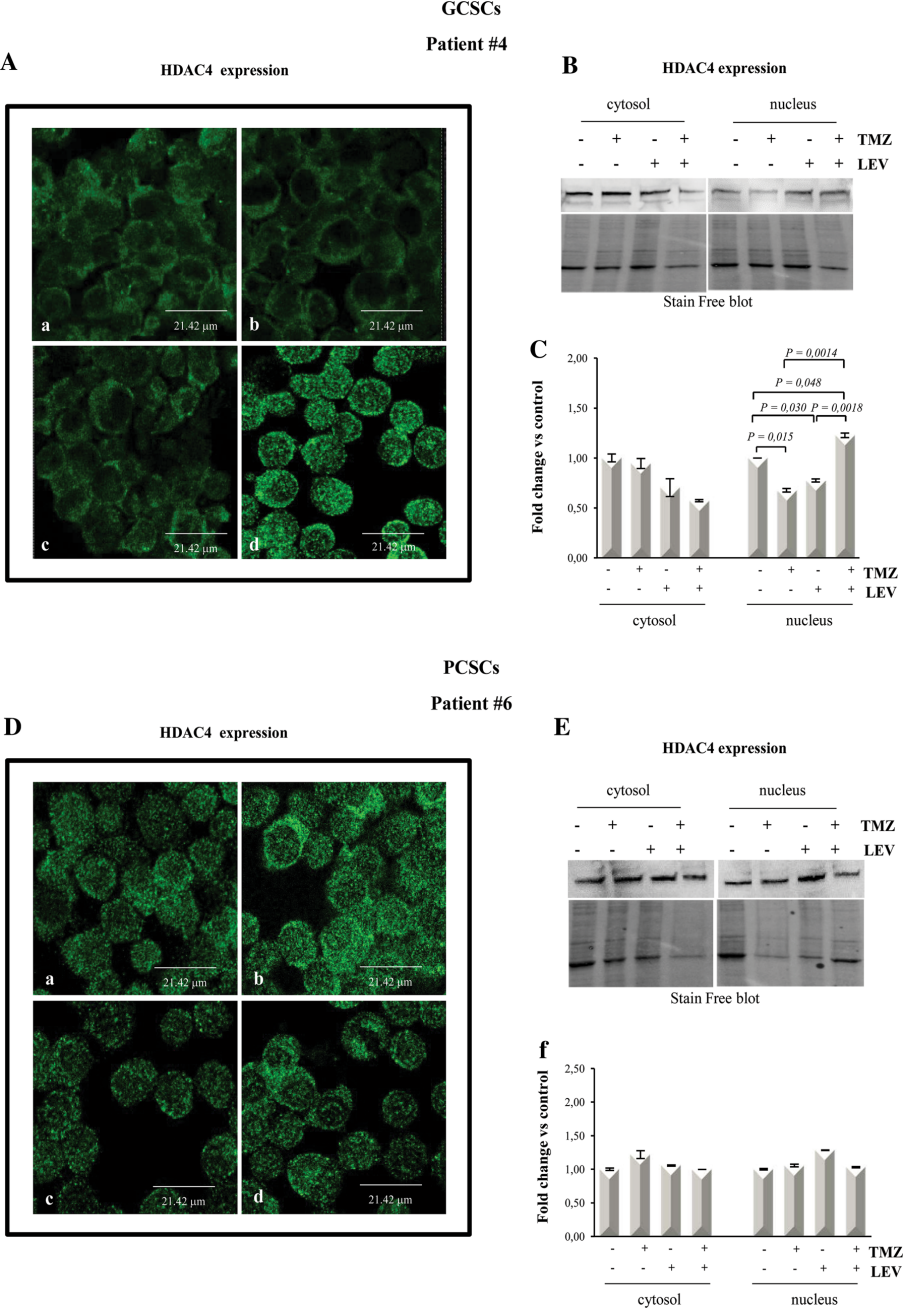
LEV effect on HDAC4 expression in GCSCs and PCSCs. GCSCs from patient #4 and PCSCs from patient #6 were treated with TMZ (250 µM), LEV (40 μg/mL) and a combination of TMZ and LEV for 48 h. **A** panels a–d HDAC4 expression was evaluated in GCSCs from patient #4 by immunofluorescence analysis. Confocal microscopy micrographs showing HDAC4 (green) in untreated control cells (a), TMZ treated cells (b), LEV treated cells (c) and LEV + TMZ treated cells (d). **B** Cytoplasmic and nuclear extracts from GCSCs (patient #4) were evaluated for HDAC4 expression by Western blot analysis. A representative immunoblot is shown. **C** Densitometric analysis of HDAC4 expression levels of three indipendent experiments normalized using Stain free technology. Statistical analysis was performed using Student’s *t*-test. P value < 0.05 was considered statistically significant. **D** panels a–d HDAC4 expression was evaluated in PCSCs from patient #6 by immunofluorescence analysis. Confocal microscopy micrographs showing HDAC4 (green) in untreated control cells (a), TMZ treated cells (b), LEV treated cells (c) and LEV + TMZ treated cells (d). **E** Cytoplasmic and nuclear extracts from PCSCs (patient #6), were evaluated for HDAC4 expression by Western blot analysis. A representative immunoblot is shown. **F** Densitometric analysis of HDAC4 expression levels of three indipendent experiments normalized using Stain free technology. Statistical analysis was performed using Student’s *t*-test. P value < 0.05 was considered statistically significant

To this purpose, we first analyzed the expression levels and the cellular localization of HDAC4 in the GCSCs derived from patient #4, that, as we previously demonstrated, greatly reduced their proliferative rate following the combined treatment with TMZ and LEV, in comparison with the PCSCs derived from patient #6, that, by contrary, showed resistance to the same treatment. Immunofluorescence analysis (Fig. 6A) and Western blotting of nuclear and cytosolic fractions (Fig. 6B, C) revealed that the combination of TMZ and LEV induced the accumulation of HDAC4 into the nucleus of GCSCs derived from patient #4. Indeed HDAC4 expression was low and diffuse in the control and in correspondence of the separate treatments (Fig. 6A: panel a, b and c) while a strong nuclear expression was detected in TMZ + LEV treated cells (Fig. 6: panel d).

Interestingly, none of the treatments induced HDAC4 nuclear translocation in PCSCs derived from patient #6 as demonstrated by the immunofluorescence and Western Blotting of nuclear and cytosolic fractions, as shown in Fig. 6D–F. We also performed Western blot analysis of HDAC4 expression in the total lysate of the PCSCs derived from patient #2 subjected to the same treatments described above, demonstrating that the low increase in HDAC4 expression observed after TMZ treatment was not significantly modified by the combined treatment with TMZ and LEV (data not shown).

### LEV induced apoptosis in TMZ-treated GCSCs but not in PCSCs

**Fig. 7:**
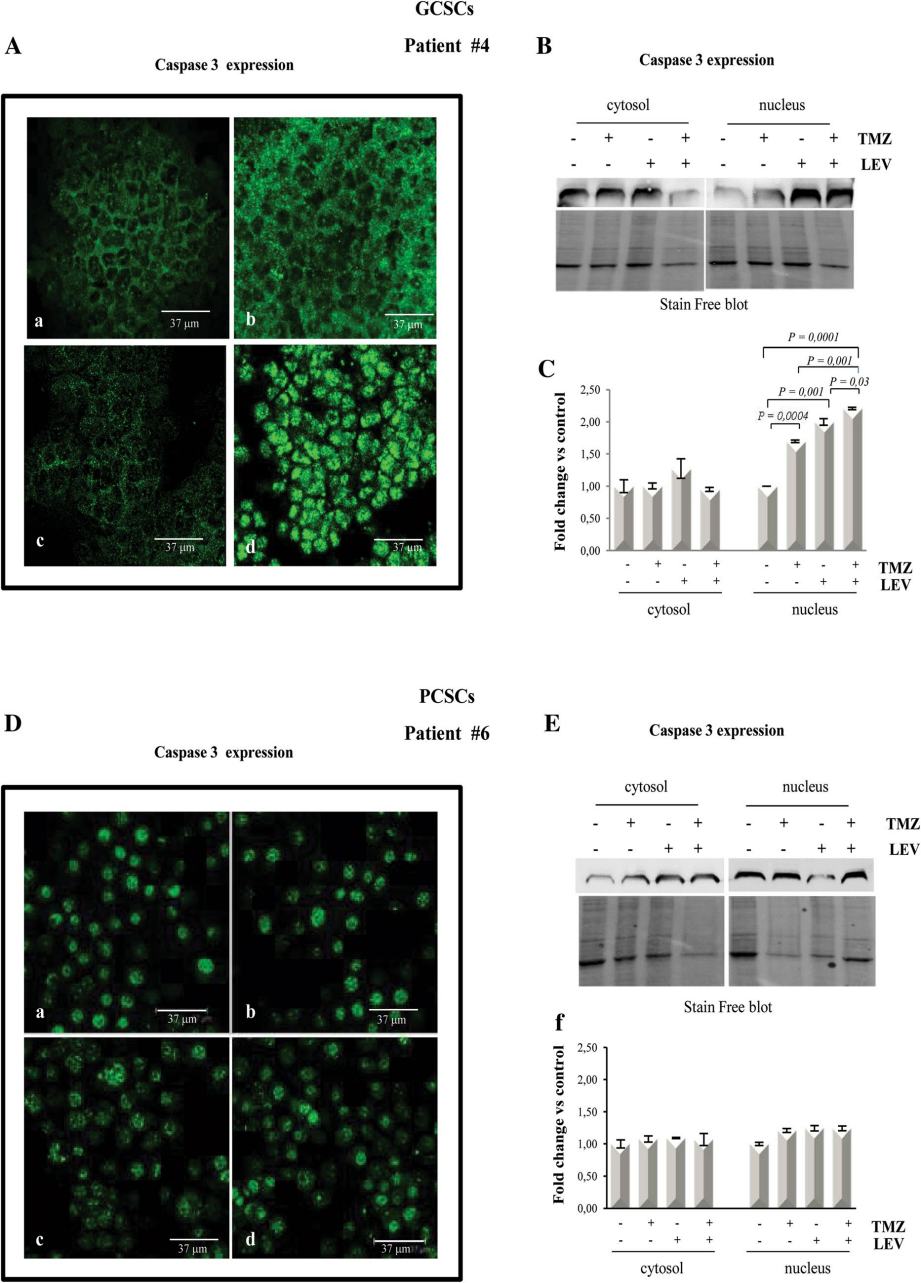
LEV effect on Caspase-3 expression in GCSCs and PCSCs. GCSCs from patient #4 and PCSCs from patient #6 were treated with TMZ (250 µM), LEV (40 μg/mL) and a combination of TMZ and LEV for 48 h. **A** panels a–d Caspase-3 expression was evaluated in GCSCs from patient #4 by immunofluorescence analysis. Confocal microscopy micrographs showing Caspase-3 (green) in untreated control cells (a), TMZ treated cells (b), LEV treated cells (c) and LEV + TMZ treated cells (d). **B** Cytoplasmic and nuclear extracts from GCSCs (patient #4) were evaluated for Caspase-3 expression by Western blot analysis. A representative immunoblot is shown. **C** Densitometric analysis of Caspase-3 expression levels of three indipendent experiments normalized using Stain free technology. Statistical analysis was performed using Student’s *t*-test. P value < 0.05 was considered statistically significant. **D** panels a–d Caspase-3 expression was evaluated in PCSCs from patient #6 by immunofluorescence analysis. Confocal microscopy micrographs showing Caspase -3 (green) in untreated control cells (a), TMZ treated cells (b), LEV treated cells (c) and LEV + TMZ treated cells (d). **E** Cytoplasmic and nuclear extracts from PCSCs (patient #6) were evaluated for Caspase-3 expression by Western blot analysis. A representative immunoblot is shown. **F** Densitometric analysis of Caspase-3 expression levels of three indipendent experiments normalized using Stain free technology. Statistical analysis was performed using Student’s *t*-test. P value < 0.05 was considered statistically significant

It has been demonstrated that one of the mechanisms involved in the cytotoxic effect of chemotherapeutic agents is apoptosis. Indeed, in Jurkat cells, all the chemotherapeutic treatments significantly induced high levels of the activity of some of the pro-caspases that are known to act as initiators (such as Caspases-2, -8 and -9) and some of the caspases (such as Caspase-3 and -6) that are known to act as effectors of apoptosis (IRI treatment only did not significantly induce the activity of Caspase-6) (Additional file 1 and Additional file 3: Figure S2). To explore whether the anti-proliferative effect of the combined treatment with TMZ and LEV was associated with apoptotic death, we tested for the presence and cellular localization of cleaved Caspase-3 under the same condition described above. By immunofluorescence analysis, we observed that in the GCSCs derived from patient #4, Caspase- 3 is present in the cytoplasm of the control and of the cells that received the individual treatments with LEV or TMZ (Fig. 7A: panels a–c), while its expression was strongly increased in the nuclei when the cells received the combined treatment LEV + TMZ (Fig. 7A: panel d). To confirm this result, we assessed the levels and the intracellular localization of cleaved Caspase-3 by Western blot analysis of nuclear and cytosolic fraction. Figure 7B and C show that Caspase-3 expression was significantly increased in the nuclei of GCSCs of patient #4 when the cells were treated with TMZ and LEV together, while none of the treatments exerted Caspase-3 significant modifications in cytosolic extracts. In contrast, immunofluorescence and Western blot analysis performed on PCSCs derived from patient #6 demonstrated that although low levels of Caspase-3 were detected in both nuclei and cytosolic fractions, none of the treatments induced changes in its expression (Fig. 7D–F). Caspase- 3 expression was also evaluated by Western blot analysis in the total lysate of the PCSCs derived from patient #2. Although a slight but significant increase in the Caspase-3 expression was detectable after TMZ treatment, this effect was not modified by the combined treatment with TMZ + LEV (data not shown).

## Discussion

Glioblastoma is a very aggressive form of brain tumor particularly resistant to the standard therapies which include maximal surgical resection, followed by combined treatment with radiotherapy and chemotherapy [6]. Alkylating agents, such as TMZ and CARB, and topoisomerase inhibitors, such as ETO and IRI, although effectively improve clinical outcomes when used alone or in combination with radiotherapy, display several adverse effects [6] and their administration has not significantly changed the survival for GBM over the last years, making chemoresistance one of the biggest problems for GBM therapy [37]. Thus, it is evident the need to identify new therapeutic strategies that can increase the life spans of patients affected by GBM.

Glioblastoma stem-like cells (GSCs) represent a subpopulation within the heterogeneous tumor mass of GBM, with a high similarity with neural stem cells [6, 38]. They are characterized by elevated proliferative rate and tumorigenic capability in vivo [39, 40] and are thought to be responsible for the resistance to standard therapies [41, 42]. It has been demonstrated that the peritumor tissue is the site of tumor recurrence in 90% of the patients [19]. This area shows complex changes such as edema, increased vascularization, abnormal gene expression and presence of numerous specialized cell types [13–18], and is also the site in which a subpopulation of cancer stem-like cells (PCSCs) has been found [12]. Although both GCSCs and PCSCs express stem cell markers, they have different characteristics in terms of self-renewal and tumorigenicity, suggesting that PCSCs may have high relevance in translational research [12].

In this study, we investigated the response of IDH1-wildtype GCSCs and PCSCs derived from six patients affected by GBM to different chemotherapeutic drugs and verified the possibility to enhance their effect through the combined treatment with adjuvant molecules. The comparison of GCSCs and PCSCs behavior and the identification of the molecules involved in their differential response to the treatments may provide a further insight in the complexity of GBM-neighboring microenvironment, which plays a crucial role in tumor progression.

Since hematopoietic cells represent the primary target of chemotherapy-related adverse effects, here we first analyzed the efficacy of different chemotherapeutic drugs (TMZ, ETO, IRI and CARB) in Jurkat cells (Additional file 2: Figure S1A). As expected, all the tested antineoplastic agents dramatically decrease the proliferation of these cells. In particular, BrdU assay demonstrates that IRI and CARB have the stronger effect in decreasing proliferation while TMZ is less efficient. Western blot analysis of PCNA expression confirms that among the used compounds, TMZ exerts the lower anti-proliferative activity (Additional file 2: Figure S1B and C). This effect seems to be related to the activation of apoptotic pathway since all the chemotherapeutic drugs increased the activity of both pro- and effector-caspases (Additional file 3: Figure S2). We then investigated the effects of the different chemotherapeutic treatments on the proliferation rate of GCSCs and PCSCs. Both cell populations resulted more resistant than Jurkat cells to the different treatments and we noted that all the chemotherapeutic agents exerted significant effects only in the cells with higher values of BrdU incorporation (GCSCs and PCSCs derived from patients #1, #2 and #3), whereas none of the drugs affected significantly the cells with a lower proliferation rate (GCSCs and PCSCs derived from patients #4, #5 and #6). Moreover, although no significant differences  have been found between GCSCs and PCSCs, TMZ is the drug with the lower efficacy in decreasing the proliferation of neurosphere clones derived from GBM of all the six patients. TMZ is a small molecule that is readily absorbed in the digestive tract and, because of its lipophilia, it is able to cross the blood–brain barrier. TMZ is the most widely chemotherapeutic drug used in patients with GBM, although the majority of the patients demonstrate de novo or acquired resistance, with subsequent tumor progression [5, 43]. Thus, the identification of the mechanisms of resistance and the attempting to enhance its effect can represent a good therapeutic strategy. MGMT repairs cytotoxic DNA lesions generated by TMZ. Many studies have shown a mechanistic link between MGMT activity and TMZ resistance, with suppression of MGMT activity resulting in increased cytotoxicity and MGMT protein overexpression which lead to resistance [31]. However, the role of MGMT in the evolution of acquired resistance is not well established as demonstrated by several papers revealing that MGMT expression is not always related to resistance to TMZ treatment [44–46]. Since our results demonstrate that TMZ is the less efficient chemotherapeutic agent in decreasing the proliferation of both GCSCs and PCSCs, we have investigated the possibility of increasing its anti-tumor activity by means of the combined use with LEV. LEV is a relatively new non-enzyme inducing AED strongly recommended as a first line drug for patients with brain tumors [47]. Recently, a growing body of evidence suggests that selected AEDs could lead to significant pharmaco-epigenetic interactions. It has been reported that LEV could act by favoring the recruitment of inhibitory complex, including HDAC, on the MGMT promoter, thus reducing its transcription [25]. Here we show that in the GCSCs the combined treatment with LEV and TMZ decreases the MGMT expression levels and induces the nuclear translocation of HDAC4, suggesting, in agreement to what reported by Bobustuc et al. [25], an HDAC4-dependent inhibitory role for MGMT transcription, thus increasing the sensitivity of these cells to TMZ treatment. In contrast, HDAC4 is expressed, although at low levels, in PCSCs, but none of the treatments modulated its expression and cellular localization. As GSCs show intrinsic deregulation in apoptotic cell death, we investigated whether in our system, the decreased proliferation rate observed in the presence of the combined treatment with LEV and TMZ could be associated with the activation of apoptotic pathway. Here we show an increased caspase 3 nuclear accumulation in GCSCs, while no change of its expression was observed in PCSCs. This result is supported by the analysis of pro-and effector-caspase expression evaluated in Jurkat cells after exposure to different chemotherapeutic agents showing that all the treatments significantly induced high levels of the activity of both classes of caspase compared to untreated cells (Additional file 3: Figure S2). All these data suggest that activation of apoptotic pathway is involved in the strong anti-proliferative effect exerted by the used antineoplastic drugs.

Taken together our results demonstrate that LEV enhances the TMZ effect on GCSCs by HDAC4-dependent downregulation of MGMT and by the activation of apoptotic pathways. PCSCs seem to be more resistant to the treatment, suggesting that the peritumoral microenvironment can favor the activation of survival mechanisms that make this therapeutic approach less effective. Our results are supported by data reported by Kim et al. demonstrating that the median progression-free survival and overall survival for patients who received LEV in combination with TMZ were significative longer than those for patients who did not receive LEV [48]. In addition, a case report was published by Peddi et al. where a continuous regression of GBM was noted in a patient who received LEV and Dexamethasone without any cancer-targeted therapy, suggesting that the response may be related to Dexamethasone and/or LEV treatment [49]. Although more studies are needed to better evaluate the role of LEV and its molecular mechanism of action, these papers together with our results strongly suggest the beneficial effect of LEV as a chemosensitizer agent.

## Conclusions

Although further studies are necessary to better characterize the GSC environment, our results suggest that the clinical therapeutic efficacy of TMZ in GBM might be potentiated by the combination treatment with LEV and that the enhancement of apoptotic pathways may represent a primary goal in the development of new and more effective strategies.

## Additional files

**Additional file 1.** Description (Results, Material and Methods) of additional figures S1 and S2.

**Additional file 2: Figure S1.** Effects of different chemotherapeutic drugs on Jurkat cell proliferation. (A) BrdU cell proliferation assay of Jurkat cells treated for 48 h with TMZ (250 µM), ETO (10 µM), IRI (10 µg/ml) and CARB (10 µg/ml). (B) Western blot analysis of total lysates from Jurkat cells, treated as described above was performed to detect PCNA expression levels; β-actin was used as a loading control. (C) Densitometric analysis of three independent experiments on PCNA expression levels. ** p< 0.01, ***p < 0001 vs control by Student’s *t*-test.

**Additional file 3: Figure S2.** Effects of different chemotherapeutic agents on apoptosis induction in Jurkat cells. Jurkat cells were treated with the same concentrations of the antineoplastic drugs described in additional Fig. 1. After 48 h, the activity of the pro-caspases-2, -8 and -9 and of the effector caspases -3 and -6 was measured by using *ApoTarget* Caspase Colorimetric Protease Assay. The results are representative of three independent experiments. *p < 0.05, **p < 0.01, ***  p< 0.001 vs control by Student’s *t*-test.

## Abbreviations

GBM: gliobastoma multiforme
CSCs: cancer stem cells
GSCs: glioblastoma stem-like cells
GCSCs: glioblastoma cancer stem cells
PCSCs: peritumoral cancer stem cells
TMZ: temozolomide
HDACs: histone deacetylases
HATs: histone acetyltransferases
MGMT: O6-methylguanine-DNA methyltransferase
CTR: control
LEV: levetiracetam
AEDs: antiepileptic drugs
PCNA: proliferating cell nuclear antigen
ETO: ETO
IRI: irinotecan
CARB: CARB
DAPI: 4′,6-diamidino-2-phenylindole

## References

[1] Ohgaki H, Kleihues P (2013). The definition of primary and secondary glioblastoma. Clin Cancer Res.

[2] Singh SK, Hawkins C, Clarke ID, Squire JA, Bayani J, Hide T (2004). Identification of human brain tumour initiating cells. Nature.

[3] Cheray M, Begaud G, Deluche E, Nivet A, Battu S, Lalloue F (2017). Cancer stem-like cells in glioblastoma.

[4] Avgeropoulos NG, Batchelor TT (1999). New treatment strategies for malignant gliomas. Oncologist..

[5] Stupp R, Mason WP, van den Bent MJ, Weller M, Fisher B, Taphoorn MJ (2005). Radiotherapy plus concomitant and adjuvant temozolomide for glioblastoma. N Engl J Med.

[6] Minniti G, Muni R, Lanzetta G, Marchetti P, Enrici RM (2009). Chemotherapy for glioblastoma: current treatment and future perspectives for cytotoxic and targeted agents. Anticancer Res.

[7] Mirimanoff RO, Gorlia T, Mason W, Van den Bent MJ, Kortmann RD, Fisher B (2006). Radiotherapy and temozolomide for newly diagnosed glioblastoma: recursive partitioning analysis of the EORTC 26981/22981-NCIC CE3 phase III randomized trial. J Clin Oncol.

[8] Hart MG, Garside R, Rogers G, Stein K, Grant R. Temozolomide for high grade glioma. Cochrane Database Syst Rev. 2013; 30:CD007415. 10.1002/14651858.CD007415.pub2 PMC6457743 23633341

[9] Piccirillo SG, Combi R, Cajola L, Patrizi A, Redaelli S, Bentivegna A, Baronchelli S (2009). Distinct pools of cancer stem-like cells coexist within human glioblastomas and display different tumorigenicity and independent genomic evolution. Oncogene.

[10] Glas M, Rath BH, Simon M, Reinartz R, Schramme A, Trageser D (2010). Residual tumor cells are unique cellular targets in glioblastoma. Ann Neurol.

[11] Piccirillo SG, Dietz S, Madhu B, Griffiths J, Price SJ, Collins VP, Watts C (2012). Fluorescence-guided surgical sampling of glioblastoma identifies phenotypically distinct tumour-initiating cell populations in the tumour mass and margin. Br J Cancer.

[12] Lama G, Mangiola A, Proietti G, Colabianchi A, Angelucci C, D’ Alessio A (2016). Progenitor/Stem Cell Markers in Brain Adjacent to Glioblastoma: GD3 Ganglioside and NG2 Proteoglycan Expression. J Neuropathol Exp Neurol.

[13] Bakshi A, Nag TC, Wadhwa S, Mahapatra AK, Sarkar C (1998). The expression of nitric oxide synthases in human brain tumours and peritumoral areas. J Neurol Sci.

[14] Roy S, Sarkar C (1989). Ultrastructural study of micro-blood vessels in human brain tumors and peritumoral tissue. J Neurooncol.

[15] Charles NA, Holland EC, Gilbertson R, Glass R, Kettenmann H (2012). The brain tumor microenvironment. Glia..

[16] Lorger M (2012). Tumor microenvironment in the brain. Cancers.

[17] Fazi B, Felsani A, Grassi L, Moles A, D’Andrea D, Toschi N (2015). The transcriptome and miRNome profiling of glioblastoma tissues and peritumoral regions highlights molecular pathways shared by tumors and surrounding areas and reveals differences between short-term and long-term survivors. Oncotarget..

[18] Mangiola A, Saulnier N, De Bonis P, Orteschi D, Sica G, Lama G (2013). Gene expression profile of glioblastoma peritumoral tissue: an ex vivo study. PLoS ONE..

[19] Park I, Tamai G, Lee MC, Chuang CF, Chang SM, Berger MS (2007). Patterns of recurrence analysis in newly diagnosed glioblastoma multiforme after three-dimensional conformal radiation therapy with respect to pre-radiation therapy magnetic resonance spectroscopic findings. Int J Radiat Oncol Biol Phys.

[20] Barciszewska AM, Gurda D, Glodowicz P, Nowak S, Naskret-Barciszewska MZ (2015). A new epigenetic mechanism of temozolomide action in glioma cells. PLoS ONE..

[21] Alonso MM, Gomez-Manzano C, Bekele BN, Yung WK, Fueyo J (2007). Adenovirus-based strategies overcome temozolomide resistance by silencing the O6-methylguanine-DNA methyltransferase promoter. Cancer Res.

[22] Nakada M, Furuta T, Hayashi Y, Minamoto T, Hamada J (2012). The strategy for enhancing temozolomide against malignant glioma. Front Oncol..

[23] Nishikawa R (2010). Standard therapy for glioblastoma-a review of where we are. Neurol Med Chir.

[24] Zhao YH, Wang ZF, Cao CJ, Weng H, Xu CS, Li K (2018). The clinical significance of O6-methylguanine-dna methyltransferase promoter methylation status in adult patients with glioblastoma: a meta-analysis. Front Neurol..

[25] Marampon F, Megiorni F, Camero S, Crescioli C, McDowell HP, Sferra R (2017). HDAC4 and HDAC6 sustain DNA double strand break repair and stem-like phenotype by promoting radioresistance in glioblastoma cells. Cancer Lett.

[26] Adamopoulou E, Naumann U (2013). HDAC inhibitors and their potential applications to glioblastoma therapy. Oncoimmunology..

[27] Witt O, Deubzer HE, Milde T, Oehme I (2009). HDAC family: what are the cancer relevant targets?. Cancer Lett.

[28] Bobustuc GC, Baker CH, Limaye A, Jenkins WD, Pearl G, Avgeropoulos NG, Konduri SD (2010). Levetiracetam enhances p53-mediated MGMT inhibition and sensitizes glioblastoma cells to temozolomide. Neuro Oncol..

[29] Louis DN, Perry A, Reifenberger G, von Deimling A, Figarella-Branger D, Cavenee WK (2016). The 2016 World Health Organization classification of tumors of the central nervous system: a summary. Acta Neuropathol.

[30] Horbinski C, Kofler J, Kelly LM, Murdoch GH, Nikiforova MN (2009). Diagnostic use of IDH1/2 mutation analysis in routine clinical testing of formalin-fixed, paraffin-embedded glioma tissues. J Neuropathol Exp Neurol.

[31] Caldera V, Mellai M, Annovazzi L, Piazzi A, Lanotte M, Cassoni P, Schiffer D (2011). Antigenic and genotypic similarity between primary glioblastomas and their derived neurospheres. J Oncol..

[32] Pallini R, Ricci-Vitiani L, Banna GL, Signore M, Lombardi D, Todaro M (2008). Cancer stem cell analysis and clinical outcome in patients with glioblastoma multiforme. Clin Cancer Res.

[33] Binda E, Visioli A, Giani F, Lamorte G, Copetti M, Pitter KL (2012). The EphA2 receptor drives self-renewal and tumorigenicity in stem-like tumor-propagating cells from human glioblastomas. Cancer Cell.

[34] Balss J, Meyer J, Mueller W, Korshunov A, Hartmann C, von Deimling A (2008). Analysis of the IDH1 codon 132 mutation in brain tumors. Acta Neuropathol.

[35] Esteller M, Hamilton SR, Burger PC, Baylin SB, Herman JG (1999). Inactivation of the DNA repair gene O6-methylguanine-DNA methyltransferase by promoter hypermethylation is a common event in primary human neoplasia. Cancer Res.

[36] Zeuner A, Pedini F, Signore M, Testa U, Pelosi E, Peschle C (2003). Stem cell factor protects erythroid precursor cells from chemotherapeutic agents via up-regulation of BCL-2 family proteins. Blood.

[37] Sarkaria JN, Kitange GJ, James CD, Plummer R, Calvert H, Weller M, Wick W (2008). Mechanisms of chemoresistance to alkylating agents in malignant glioma. Clin Cancer Res.

[38] Sanai N, Alvarez-Buylla A, Berger MS (2005). Neural stem cells and the origin of gliomas. N Engl J Med.

[39] Ignatova TN, Kukekov VG, Laywell ED, Suslov ON, Vrionis FD, Steindler DA (2002). Human cortical glial tumors contain neural stem-like cells expressing astroglial and neuronal markers in vitro. Glia..

[40] Singh SK, Clarke ID, Terasaki M, Bonn VE, Hawkins C, Squire J, Dirks PB (2003). Identification of a cancer stem cell in human brain tumors. Cancer Res.

[41] Chen J, Li Y, Yu TS, McKay RM, Burns DK, Kernie SG, Parada LF (2012). A restricted cell population propagates glioblastoma growth after chemotherapy. Nature.

[42] Reya T, Morrison SJ, Clarke MF, Weissman IL (2001). Stem cells, cancer, and cancer stem cells. Nature.

[43] Burton EC, Prados MD (2000). Malignant gliomas. Curr Treat Options Oncol.

[44] Pandith AA, Qasim I, Zahoor W, Shah P, Bhat AR, Sanadhya D, Shah ZA, Naikoo NA (2018). Concordant association validates MGMT methylation and protein expression as favorable prognostic factors in glioma patients on alkylating chemotherapy (Temozolomide). Sci Rep..

[45] Kitange GJ, Carlson BL, Schroeder MA, Grogan PT, Lamont JD, Decker PA, Wu W, James CD, Sarkaria JN (2009). Induction of MGMT expression is associated with temozolomide resistance in glioblastoma xenografts. Neuro Oncol..

[46] Blough MD, Westgate MR, Beauchamp D, Kelly JJ, Stechishin O, Ramirez AL, Weiss S, Cairncross JG (2010). Sensitivity to temozolomide in brain tumor initiating cells. Neuro Oncol..

[47] van Breemen MS, Wilms EB, Vecht CJ (2007). Epilepsy in patients with brain tumours: epidemiology, mechanisms, and management. Lancet Neurol..

[48] Kim YH, Kim T, Joo JD, Han JH, Kim YJ, Kim IA, Yun CH, Kim CY (2015). Survival benefit of levetiracetam in patients treated with concomitant chemoradiotherapy and adjuvant chemotherapy with temozolomide for glioblastoma multiforme. Cancer.

[49] Peddi P, Ajit NE, Burton GV, El-Osta H (2016). Regression of a glioblastoma multiforme: spontaneous versus a potential antineoplastic effect of dexamethasone and levetiracetam. BMJ Case Rep..

