# Supplementary material for: Levetiracetam enhances the temozolomide effect on glioblastoma stem cell proliferation and apoptosis
Source: Cancer Cell Int. 2018 Sep 10;18:136. doi: 10.1186/s12935-018-0626-8 (PMC6131782; doi:10.1186/s12935-018-0626-8)
Supplement: Supplementary file 1 — Additional file 1. Description (Results, Material and Methods) of additional figures S1 and S2. [file 12935_2018_626_MOESM1_ESM.docx]

**ADDITIONAL FILE**

**Results**

**Effect of chemotherapeutic treatments on Jurkat cells proliferation.**

Since hematopoietic cells represent a good general model of chemotherapeutic agent activity, we first analyzed the efficacy of different chemotherapeutic drugs in Jurkat cells. To this purpose Jurkat cells were treated with TMZ (250 µM), ETO (10 µM), IRI (10 µg/ml) and CARB (10 µg/ml), for 48 hours and then the proliferation rate was evaluated by BrdU cell proliferation assay. As shown in additional Fig.1A, all the used chemotherapeutic agents exerted a significant reduction in BrdU incorporation. In particular, our results demonstrated that TMZ decreased proliferation by 52%, ETO by 53%, while IRI and CARB had the stronger effect since they decreased BrdU incorporation by 67%, versus untreated cells at the end of the culture. The effect of chemotherapeutic agents in Jurkat cells proliferation has been also evaluated by Western Blot analysis of the expression of the proliferating marker PCNA (Additional Fig.1B). According with the BrdU assay, densitometric analysis (Additional Fig.1C) revealed that although PCNA expression was severely affected by all the used chemotherapeutic agents, TMZ had the lower effect.

**Induction of apoptosis by different chemotherapeutic agents in Jurkat cells.**

It has been demonstrated that one of the mechanisms involved in the cytotoxic effect of chemotherapeutic agents is apoptosis. To verify whether the chemotherapeutic-dependent reduction of Jurkat cell proliferation was associated with apoptotic death, we analyzed the activity of some of the pro-caspases that are known to act as initiators (such as caspases-2, -8 and -9) and some of the caspases (such as caspase-3 and -6) that are known to act as effectors of apoptosis. Jurkat cells were treated for 48 hours with the same concentrations of the antineoplastic drugs described above and then the caspase colorimetric assay was performed. Additional Fig.2 shows that all the chemotherapeutic treatments significantly induced high levels of the activity of both classes of caspases compared to untreated cells (IRI treatment only did not significantly induce the activity of caspase 6). These results suggest that activation of apoptotic pathway is involved in the strong anti-proliferative effect exerted by the used antineoplastic drugs on Jurkat cells.

**Methods**

**Cell cultures**

Jurkat cells are routinely cultured in RPMI 1640 medium (Life Technologies [GIBCO], Burlington, ON, Canada) supplemented with 10% FCS (Wisent, St-Bruno, QC, Canada), 10 mM Hepes and 100 units/mL penicillin/streptomycin (both from Life Technologies). Cell cultures were maintained at 37°C in a 5% CO2 humidified atmosphere.

**BrdU Cell Proliferation Assay**

Jurkat cells were seeded at the density of 50.000 cells/well in 96-well plates and incubated overnight. The cells were then treated with the appropriate chemotherapeutic agents, as indicated in the additional Figure 1 for 48 hours. Finally, 10 µM BrdU was added to the plates and the cells were incubated overnight. BrdU proliferation assay was performed according to the manufacturer’s instructions (Cell Signaling, #6813 Danvers, MA, USA).

**Quantitative determination of caspase activity in Jurkat cells**

Quantitative analysis of caspases proteolytic activity in Jurkat cells was determined by the Apo*Target* Caspase Colorimetric Protease Assay Sampler kit (Caspases-2, -3, -6, -8, -9) (#KHZ1001, Invitrogen, Carlsbad, CA, USA). Shortly, the apoptosis was induced by the addition of above mentioned antineoplastic drugs for 48 hours and then the cells were suspended in Cell Lysis Buffer. The cytosol extracts were processed according to the manufacturer’s instructions. Fold-increase in caspase -2, -3, -6, -8, -9 activity was evaluated in a 405 nm microplate reader (Additional Figure 2).

**Western Blot Analysis**

For immunoblotting analysis, Jurkat cells were washed in 1x PBS, harvested and lysed in 1x Cell Lysis Buffer (Cell Signaling #9803) containing 1mM PMSF (Cell Signaling #8553) and a complete protease inhibitor cocktail (Cell Signaling #5872) for 30 min at 4°C. Then the cells were sonicated briefly and the extracts were centrifuged 10 minutes at 14,000 x g in a cold microfuge. Protein concentration was determined by Bradford Protein Assay (Bio-Rad Laboratories Inc, Hercules,CA, USA) according to the manufacturer’s instructions. Equal amounts of proteins were then separated by SDS/ PAGE (4–20% Mini-PROTEAN® TGX™ Precast Protein Gels, Bio-Rad Laboratories Inc.) and transferred to a nitrocellulose membrane (GE Healthcare, Piscataway, NJ, USA). Membranes were blocked with Tris-buffered saline (TBS) 1X (Bio-Rad Laboratories Inc.) supplemented with 0.1% Tween-20 and containing 5% nonfat milk for 1 hour at room temperature (RT). The primary antibody used in this work was anti-PCNA (1:1000, mouse monoclonal antibody, M0879, Dako, Santa Clara, CA, USA); Blots were then incubated with horseradish peroxidase-conjugated secondary antibody (1:10000, Vector Laboratories, Burlingame, CA, USA) for 1 h RT. Signals were captured by ChemiDoc™ Imaging System (Bio-Rad Laboratories, Hercules, CA, USA) using an enhanced chemiluminescence system (SuperSignal Chemoluminescent substrate, Thermo Fisher Scientific Inc. Waltham, MA, USA) and densitometric analyses were performed with Image Lab™ Touch Software (Bio-Rad Laboratories). All experiments were carried out in triplicate and representative results are shown.

**Statistical Analysis**

Each experiment was repeated three times. Data are presented as the mean + SD. Statistical analysis was performed using the Student’s *t*-test, assuming equal variance, and p-values were calculated based on the 2-tailed test. P-value of < 0.05 was considered statistically significant.
