# Supplementary figures and images for: Levetiracetam enhances the temozolomide effect on glioblastoma stem cell proliferation and apoptosis
Source: Cancer Cell Int. 2018 Sep 10;18:136. doi: 10.1186/s12935-018-0626-8 (PMC6131782; doi:10.1186/s12935-018-0626-8)

**A**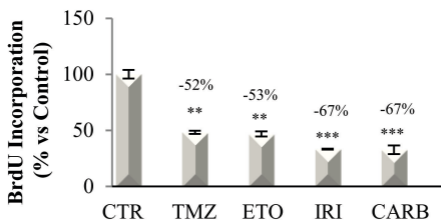**B**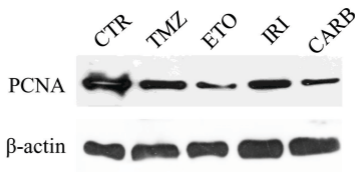**C**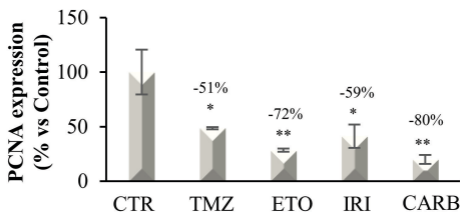**Additional Figure 1**

Supplement: Supplementary file 2 — Additional file 2: Figure S1. Effects of different chemotherapeutic drugs on Jurkat cell proliferation. (A) BrdU cell proliferation assay of Jurkat cells treated for 48 h with TMZ (250 µM), ETO (10 µM), IRI (10 µg/ml) and CARB (10 µg/ml). (B) Western blot analysis of total lysates from Jurkat cells, treated as described above was performed to detect PCNA expression levels; β-actin was used as a loading control. (C) Densitometric analysis of three independent experiments on PCNA expression levels. ** p< 0.01, ***p < 0001 vs control by Student’s t-test. [file 12935_2018_626_MOESM2_ESM.pdf]

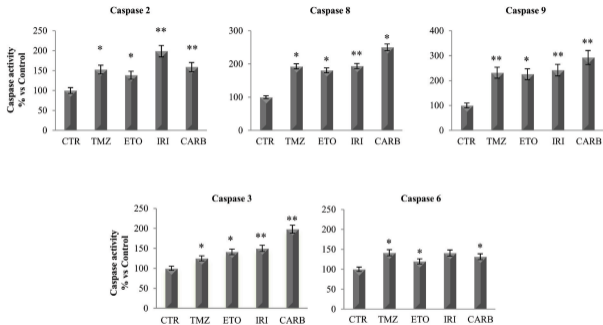

**Additional Figure 2**

Supplement: Supplementary file 3 — Additional file 3: Figure S2. Effects of different chemotherapeutic agents on apoptosis induction in Jurkat cells. Jurkat cells were treated with the same concentrations of the antineoplastic drugs described in additional Fig. 1. After 48 h, the activity of the pro-caspases-2, -8 and -9 and of the effector caspases -3 and -6 was measured by using ApoTarget Caspase Colorimetric Protease Assay. The results are representative of three independent experiments. *p < 0.05, **p < 0.01, *** p< 0.001 vs control by Student’s t-test. [file 12935_2018_626_MOESM3_ESM.pdf]
